# State-dependent μ-opioid modulation of social motivation

**DOI:** 10.3389/fnbeh.2014.00430

**Published:** 2014-12-12

**Authors:** Guro E. Loseth, Dan-Mikael Ellingsen, Siri Leknes

**Affiliations:** ^1^Department of Psychology, University of OsloOslo, Norway; ^2^Department of Physiology, University of GothenburgGothenburg, Sweden; ^3^The Intervention Centre, Oslo University HospitalOslo, Norway

**Keywords:** OPRM1, affiliative behaviors, opioids, social reward, social touch, social comfort, social support, μ-opioid

## Abstract

Social mammals engage in affiliative interactions both when seeking relief from negative affect and when searching for pleasure and joy. These two motivational states are both modulated by μ-opioid transmission. The μ-opioid receptor (MOR) system in the brain mediates pain relief and reward behaviors, and is implicated in social reward processing and affiliative bonding across mammalian species. However, pharmacological manipulation of the μ-opioid system has yielded opposite effects on rodents and primates: in rodents, social motivation is generally increased by MOR agonists and reduced by antagonists, whereas the opposite pattern has been shown in primates. Here, we address this paradox by taking into account differences in motivational state. We first review evidence for μ-opioid mediation of reward processing, emotion regulation, and affiliation in humans, non-human primates, rodents and other species. Based on the consistent cross-species similarities in opioid functioning, we propose a unified, state-dependent model for μ-opioid modulation of affiliation across the mammalian species. Finally, we show that this state-dependent model is supported by evidence from both rodent and primate studies, when species and age differences in social separation response are taken into account.

## Introduction

It is a popular belief that endorphins make us feel good. Surprisingly little evidence for this notion can be found in the scientific literature, however. It is well established that μ-opioids help us to regulate pain (Zubieta et al., 2001). The importance of the μ-opioid receptor (MOR) system for reward processes in non-human animals is also well documented (e.g., Berridge and Kringelbach, 2013; Laurent et al., 2014). In contrast, little evidence links endorphin release to the experience of positive emotion in humans (but see these molecular imaging studies; Boecker et al., 2008; Koepp et al., 2009; Hsu et al., 2013). Here, we propose a model for opioid modulation of social well-being across mammalian species. There are three main types of opioid receptors in the brain, μ-, δ - and κ-receptors, and although there is increasing evidence implicating the δ - and κ-receptors in social behavior (Lutz and Kieffer, 2013; Resendez and Aragona, 2013), this review will focus on the considerably more studied μ-opioid system. The μ-opioid receptor (MOR) system is known to interact with the dopamine system in brain regions implicated in reward processing (e.g., Hagelberg et al., 2002; Lintas et al., 2011; Colasanti et al., 2012). The MOR system is also proposed to interact with oxytocin and dopamine in social bonding and social reward processing (Depue and Morrone-Strupinsky, 2005; Tops et al., 2014). The neural processes modulating and mediating social interactions are without doubt very complex. By focusing here on state-dependent μ-opioid modulation of social motivation we aim to further the understanding of how basic reward processing influences social interactions.

**Figure 1:**
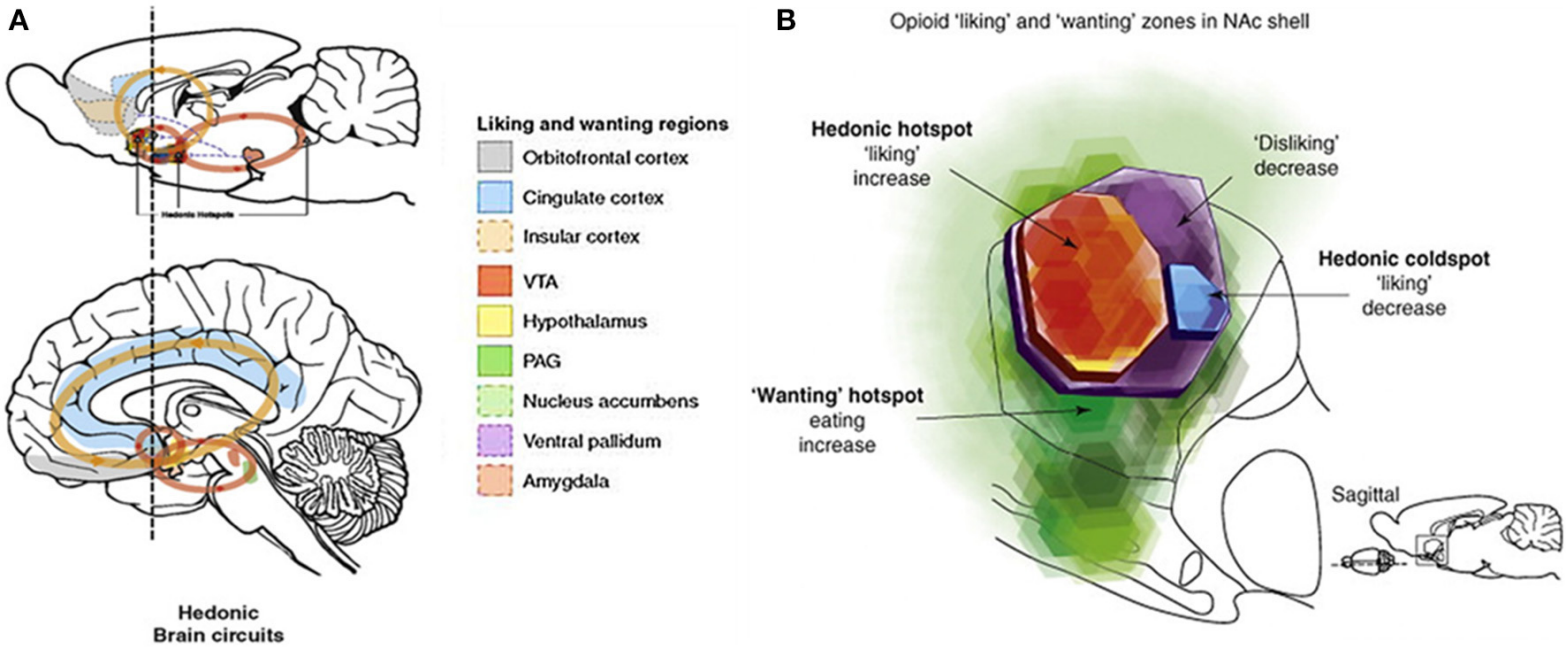
**Reward circuits in the brain. (A)** Reward related brain areas in the rodent and human brain. These circuits are densely innervated with μ-opioid receptors, in addition to dopamine receptors and receptors for other neurotransmitters (Kringelbach and Berridge, 2009). Reward can be parsed into three components: hedonic “liking,” motivational “wanting” and cognitive “learning” (Berridge and Robinson, 2003; Berridge and Kringelbach, 2008; Berridge et al., 2009), and similarly to other primary rewards such as food or drugs, social rewards create feelings of wellbeing and pleasure (“liking”); motivate approach behaviors (“wanting”) and elicit associative learning of socially relevant cues (“learning”) (Trezza et al., 2011a). Reprinted from Kringelbach and Berridge (2009), with permission from Elsevier. **(B)** Hedonic hotspots have been identified in the rodent nucleus accumbens (NAc) and ventral pallidum (VP). Microinjections of the specific μ-opioid agonist DAMGO within the hotspots generate or intensify “liking”-responses to sweet taste rewards (Berridge and Kringelbach, 2013; Castro and Berridge, 2014), while injecting DAMGO into striatal areas outside these hotspots selectively enhances “wanting” without affecting “liking” (Mahler and Berridge, 2012) similarly to what is found with dopamine injections both within and outside of the hotspots (Peciña and Berridge, 2013). Reprinted from Berridge et al. (2009), with permission from Elsevier.

Engaging in affiliative interactions such as social play and social grooming is associated with endogenous μ-opioid release in brain reward circuitry in both rodents and primates (see Figure 1). In juvenile rats, rough-and-tumble play leads to increased central μ-opioid receptor activation, as suggested by both *in vitro* (Panksepp and Bishop, 1981) and *in vivo* studies (Vanderschuren et al., 1995e). In non-human primates, social grooming can elicit endogenous μ-opioid release, as demonstrated in a study where large increases in cerebrospinal levels of β-endorphins were observed following social grooming (Keverne et al., 1989). Both social play and grooming involve a great deal of tactile contact, and facilitate social joy and comfort (for a quick introduction to the most relevant social touch behaviors mentioned in this review, see Textbox 1). Grooming-induced MOR activation is thought to be a key facilitator of long-term relationship formation and maintenance in species of non-human primates that frequently engage in mutual partner-specific social grooming (Dunbar, 2010; Machin and Dunbar, 2011). Intriguingly, a series of studies report that adult mice engage more in social grooming when they interact with siblings, and subsequently display larger μ-opioid-mediated decreases in pain sensitivity, as compared to mice interacting with unrelated cage mates (D'Amato and Pavone, 1993, 1996; D'Amato, 1998). These findings suggest that endogenous μ-opioid responses to social interactions vary according to the nature and quality of the relationship between animals engaging with each other. MOR mechanisms have long been hypothesized to play a central role in reward processes promoting and maintaining affiliation (Depue and Morrone-Strupinsky, 2005; Machin and Dunbar, 2011). However, the question of whether brain endorphin release is either a prerequisite that *causes* attachment and social motivation, or is something that follows as an *effect* of social contact and successful bonding—or perhaps both—is still being explored.

Textbox 1 **Affiliative touch behaviors in rodents and non-human primates.**

Throughout the life span of social animals, touch fosters and communicates intimacy and cooperation, and works as a tangible sign of social support (Hertenstein et al., 2006). Touch interactions such as grooming, huddling, and playing are common in most mammalian species, and aid establishment and maintenance of social, romantic, sexual and parental relationships. Here we outline the touch behaviors that are the most commonly observed in studies of μ-opioid involvement in social comfort and affiliation.

***Social grooming*** in non-human primates forms the basis of long term relationships that provide social support and protection (Dunbar, 1980; Seyfarth and Cheney, 1984). Primate grooming involves sweeping through someone else's fur with hands and lips and removing impurities—the relaxing effect can be measured physiologically, e.g., reduced heart rate (Dunbar, 2010), as well as behaviorally, e.g., reduced stress-related scratching (Schino et al., 1988). Rodent maternal licking and grooming of the pup is central to mother-infant bond formation (Broad et al., 2006) and supports the development of resilient stress regulation systems (Liu et al., 1997; Caldji et al., 1998, 2000; Weaver et al., 2004) and is also associated with sociability in adolescence (Moore and Power, 1992; Parent and Meaney, 2008; Van Hasselt et al., 2012).

**Figure:**
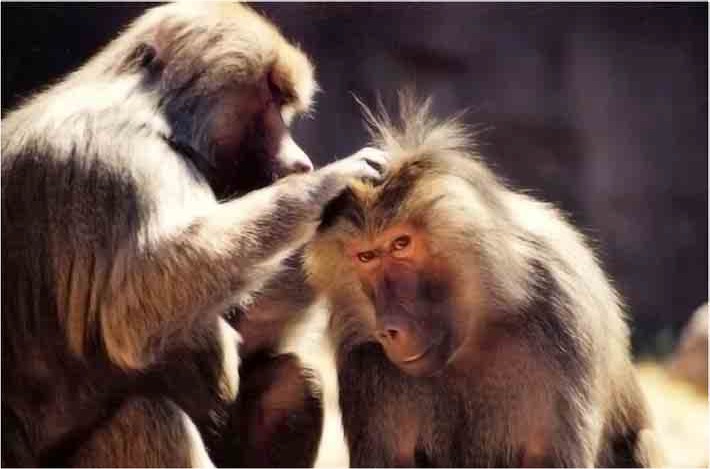
Photo by Dan Shouse is licenced under CC-BY-NC-ND 2.0.

***Huddling*** can be defined as motionless physical contact, and typically occurs when animals are resting side by side. This mediates pair bonding in adult prairie voles (Burkett et al., 2011) and sibling bonding in rat pups (Alberts, 2007); aids infant well-being in primates (Harlow and Zimmermann, 1959), and homeostatic regulation in infant rodents (Nelson and Panksepp, 1998). Rat pups respond to separation from the huddle group with distress behaviors such as ultrasonic vocalizations (USVs), which are greatly attenuated by reinstatement of physical contact (Hofer and Shair, 1987; Stanton et al., 1987; Carden and Hofer, 1992; Hofer et al., 1993).

**Figure:**
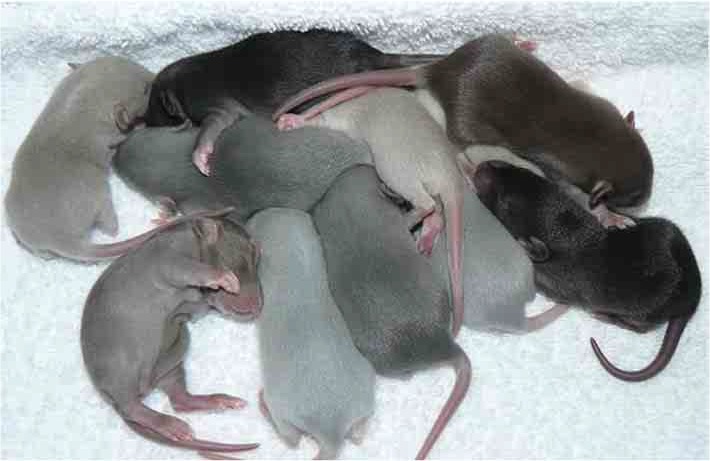
Photo by braindamaged217 is licenced under CC-BY-NC-ND 2.0.

***Social play*** involves a great deal of tactile contact, and inhibiting the sense of touch with a local anesthetic significantly reduces playing in juvenile rats (Siviy and Panksepp, 1987). Physical play interactions such as wrestling or tickling are typically accompanied by laughter in both humans and non-human primates (Vettin and Todt, 2005; Davila Ross et al., 2009) and positive USVs in rodents (Panksepp and Burgdorf, 2000, 2003; Burgdorf et al., 2005), reflecting positive play-induced affect (Bachorowski and Owren, 2001; Gervais and Wilson, 2005; Panksepp, 2007, 2011; Burgdorf et al., 2011). Similar forms of physical play are common across mammalian species, especially in juveniles (Bekoff and Byers, 1981, 1998; Fagen, 1981). Play interactions help establish social organization and interaction patterns in human and non-human primates (Maestripieri and Ross, 2004; Palagi et al., 2004; Mancini and Palagi, 2009; Cordoni and Palagi, 2011; Norscia and Palagi, 2011; Ciani et al., 2012; Shimada, 2013) and in rodents (Meaney and Stewart, 1981; Van Den Berg et al., 1999; Pellis et al., 2010).

**Figure:**
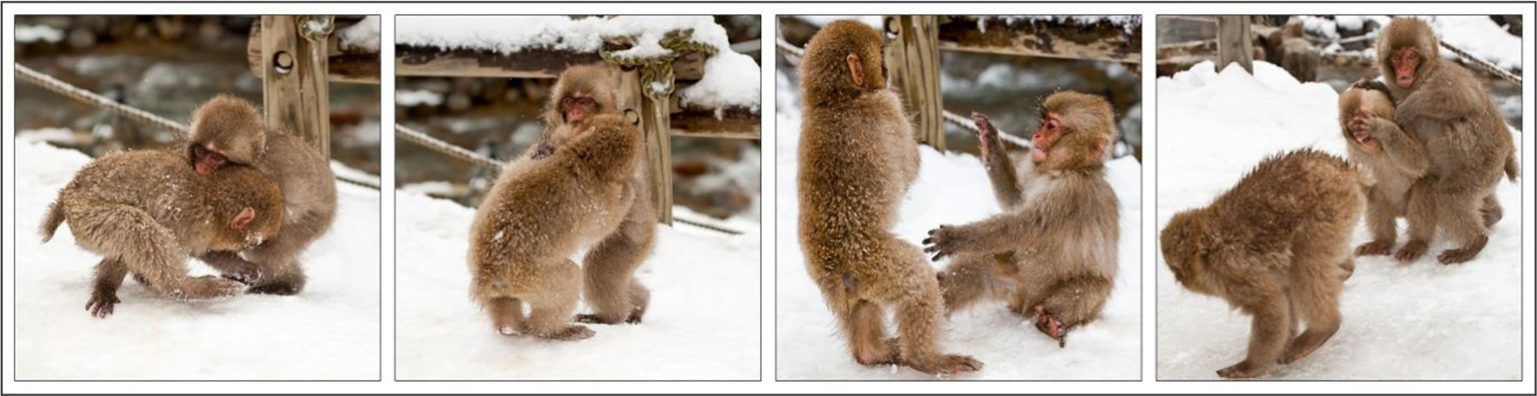
Photo by Clint Koehler is licenced under CC-BY-NC-ND 2.0.

Panksepp and colleagues first formulated the “The Brain Opioid Theory of Social Attachment” (BOTSA) theory in 1978. They argued that firstly, given the importance of social behaviors such as parental care and altruistic behaviors for survival of both the individual and the species, affiliative behaviors were likely to have evolved from more basic systems sub-serving pain perception. Secondly, several lines of evidence pointed to striking parallels between social attachment and drug addiction (Panksepp et al., 1978). Both phenomena are associated with a similar behavioral repertoire of proximity- seeking and approach behaviors, as well as profound distress and physiological stress responses to separation or deprivation that are promptly alleviated by contact restoration with the drug or object of attachment (Panksepp et al., 1978; Nelson and Panksepp, 1998; Insel, 2003). BOTSA proposes that social contact alleviates isolation distress and induces positive emotions through the release of endogenous opioids, while separation or social isolation causes opioid withdrawal symptoms and negative affect. This way, the pleasant effects of endorphin release and the aversive effects of endorphin withdrawal are thought to motivate us to seek social contact and maintain proximity to individuals we are emotionally attached to Panksepp et al. (1978, 1980b).

The first evidence supporting BOTSA came from a series of psychopharmacological studies in infants of several species, showing that μ-opioid agonists like morphine reduced and opioid antagonists like naltrexone and naloxone exacerbated distress calls and social contact seeking during and following social separation and isolation. Without apparent sedative effects, MOR agonists such as morphine profoundly reduced isolation-induced crying, while blocking the receptors with an opioid antagonist exacerbated or even *induced* separation distress behaviors in isolated puppies (Panksepp et al., 1978; Knowles et al., 1989), infant guinea pigs (Herman and Panksepp, 1978), rat pups (Panksepp et al., 1980b; Carden and Hofer, 1990b), chicks (Panksepp et al., 1980a), and infant rhesus monkeys (Kalin et al., 1988). In rat pups, socially mediated reductions in distress vocalizations were completely blocked by the opioid antagonist naltrexone (Carden and Hofer, 1990a), suggesting that social comfort was indeed mediated by the MOR system. This evidence consistently showed that across species, MOR *deactivation* increased the motivation to seek social contact for comfort in distressed infants, while MOR *activation* reduced this motivation and left the infant less distressed.

Studies of adult and juvenile non-human primates lent further support to the BOTSA postulations that exogenous opiates can replace the need for social contact, and that blocking the MOR system enhances overall motivation for social interaction. Following brief social isolation, animals injected with morphine engaged less in social grooming (Keverne et al., 1989), while those injected with μ-opioid antagonists such as naltrexone and naloxone made more solicitations and spent more time receiving grooming (Meller et al., 1980; Fabre-Nys et al., 1982; Keverne et al., 1989; Schino and Troisi, 1992; Martel et al., 1995; Graves et al., 2002).

### Opposite μ-opioid system modulation of social motivation in primates and rodents?

In contrast to the findings in infants and socially isolated adult primates, social isolation leads to increases in social play interactions in juvenile rats. This increase is further *amplified* by low-dose morphine injections, while μ-opioid antagonist treatment leads to reductions in social play (Beatty and Costello, 1982; Panksepp et al., 1985; Siegel and Jensen, 1985, 1986; Vanderschuren et al., 1995a,c,d, 1996; Trezza and Vanderschuren, 2008a,b). Similar effects have been found in adult rats, where social grooming was increased by MOR agonists and decreased by MOR antagonists (Van Ree and Niesink, 1983; Niesink and van Ree, 1984, 1989). These effects of MOR manipulation on rodent adult and juvenile social behavior appear to be at odds with those observed in primate studies where MOR agonist treatment reduced social approach behaviors. In sum, the available literature indicates that increased MOR-activation *reduces* motivation for social contact in infants of several species including rodents, as well as in adult primates, but *enhances* motivation for social contact in juvenile and adult rodents. This could indicate that the MOR system plays opposite roles in social reward for rodents and primates, and that species differences become evident through development.

In the following, we address this paradox. To investigate whether there is indeed evidence supporting opposing functions of the opioid system in primate and rodent social reward processing, we first summarize the current knowledge on μ-opioid mediation of social reward processing and emotion regulation, and on μ-opioid mediation of affiliation, in both human and non-human animals. We propose a unified, state-dependent model for μ-opioid modulation of affiliation across the mammalian species. We then elaborate on this model by presenting further evidence, focusing on how differences in motivational state may affect social motivation and responses to pharmacological MOR manipulation.

## The role of the μ-opioid system in social reward processing and emotion regulation

Pain and pleasure are core driving forces behind behavior. We generally seek to avoid punishments and to achieve rewards. When the pursuit is successful, we experience subjective feelings of relief or pleasure. When we fail, the result is displeasure. We can call the pursuit of pleasure in the absence of a stressor *pleasure seeking*; whilst behavior aimed at reducing discomfort in the presence of a stressor can be called *pain avoidance.* Both pleasure seeking and pain avoidance are reward behaviors, driven by positive and negative reinforcement, respectively (Navratilova and Porreca, 2014). The endogenous μ-opioid receptor (MOR) system modulates both pain and pleasure (Leknes and Tracey, 2008), as well as behaviors related both to food (Berridge, 1996) and to social rewards such as sex (Mahler and Berridge, 2012) or affiliation (Lutz and Kieffer, 2013). Here, we summarize evidence that reveals consistent similarities in reward-related MOR functioning between rodents and humans. These cross-species correspondences substantiate an interpretation of the paradoxical effects MOR stimulation had on social motivation in rodents and primates as caused by differences in motivational state, rather than in neurobiological functioning.

Endogenous MOR transmission contributes to the encoding of relative reward value. Laboratory rats for instance ate less of their favorite food—chocolate chip cookies—if MOR were blocked by an antagonist, but they would still eat normal amounts of standard rat chow (Cooper and Turkish, 1989). Humans show a similar decrease in preference for high-fat, high-sugar foods after MOR antagonism (Mercer and Holder, 1997; Yeomans and Gray, 1997; Adolphs and Spezio, 2006; Nathan et al., 2012). If the μ-opioid receptors are instead stimulated with a MOR agonist, rodents' intake of sweet and fatty foods increases (Zhang et al., 1998; Peciña and Berridge, 2000; Kelley et al., 2002), and so do behaviors indicative of hedonic liking such as lip licking (Peciña and Berridge, 2000). This indicates that pharmacological enhancement of MOR system activity increases pleasure seeking specifically of high-value rewards (for a review of opioid signaling and food reward, see Taha, 2010).

**Figure 2:**
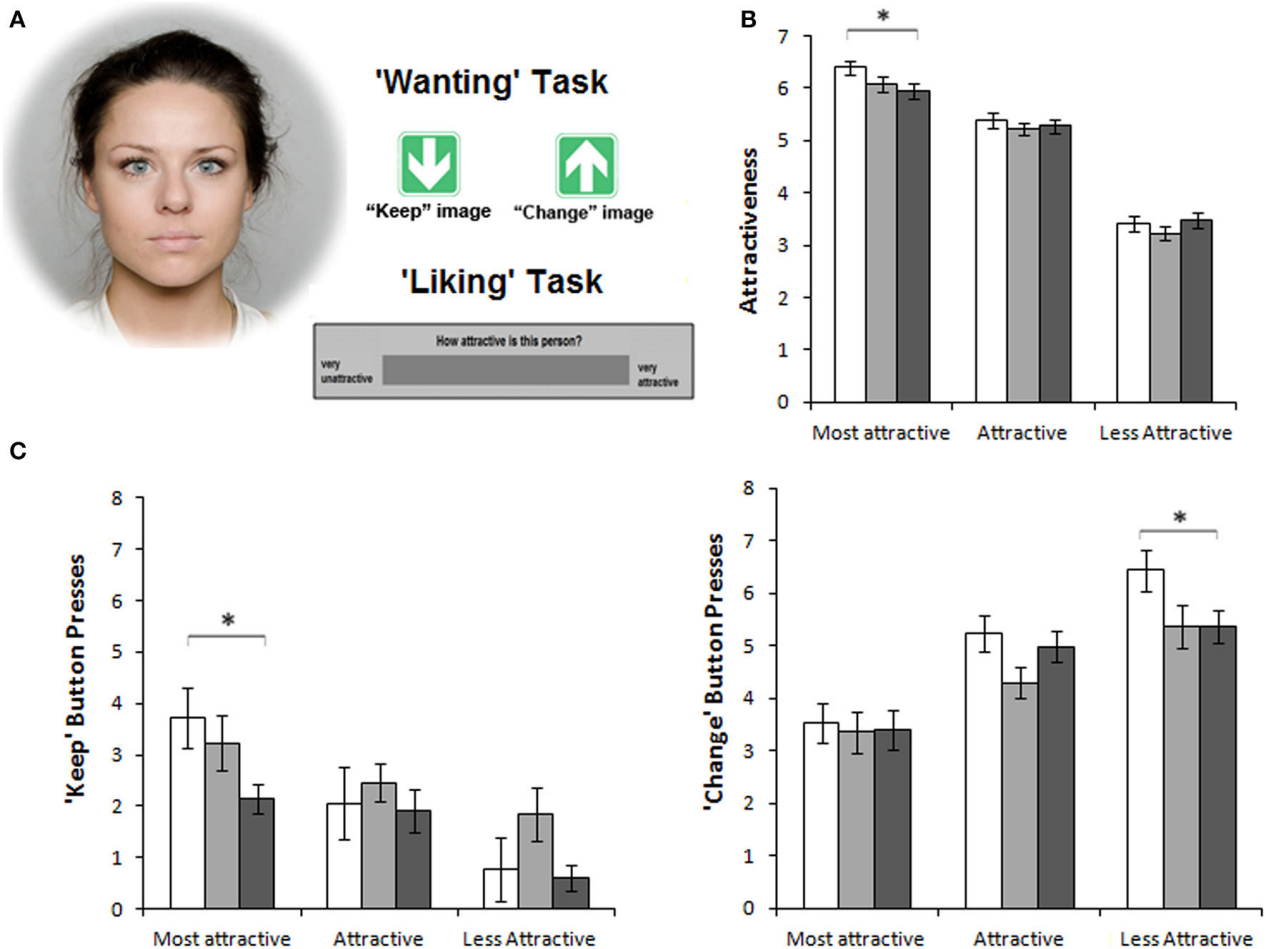
**The μ-opioid system promotes social motivation in humans**. Figure from Chelnokova et al. (2014), where thirty healthy males received a μ-opioid agonist (morphine 10 mg), a non-selective opioid antagonist (naltrexone 50 mg) or placebo (per-oral) on three separate days. Error bars represent within-subject s.e.m. Results from the morphine condition are shown in white; placebo, light gray; naltrexone, dark gray. **(A)** In two “wanting” and “liking” tasks, participants viewed pictures of faces with varying attractiveness levels. In the “wanting” task, participants could press one of two arrow keys to view the image for longer (“keep”) or shorter (“change”) time than the pre-set 5 s, without altering total task duration. In the “liking” task, participants rated attractiveness of each face using a VAS scale. **(B)** Morphine treatment enhanced and naltrexone treatment decreased men's “liking” of the most beautiful female faces (ratings on the VAS scale from 0 to 10). **(C)** “Wanting” of attractive females, as measured by the total of “keep” button presses, was similarly affected by opioid manipulations. However, morphine also increased motivation to avoid viewing the least attractive female faces, as measured by the total of “change” button presses. ^*^P < 0.05. Reprinted from Chelnokova et al. (2014) by permission from Macmillan Publishers Ltd.

A similar pattern of effects from MOR manipulations is found for sexual preference. Male rats displayed enhanced sexual “wanting” of females in oestrous after MOR stimulation, while their desire to mate with non-oestrous females remained the same (Mahler and Berridge, 2012). We recently reported an analogous finding in a laboratory study in humans. The human MOR system was manipulated with per-oral doses of an agonist (morphine) or antagonist (naltrexone). Male participants viewed pictures of female faces on a computer screen. In a *liking* task, they rated the attractiveness of the faces. In a *wanting* task, they pressed buttons to regulate the display time of each face. For both tasks, the largest effects of MOR manipulations were found for the high-reward stimuli, i.e., the most attractive female faces. Compared to placebo, morphine increased and naltrexone decreased attractiveness ratings and number of presses on a “keep-picture-on-screen”-button (Chelnokova et al., 2014; See Figure 2 for more details). Facial attractiveness is considered a signal of health and fertility, i.e., of evolutionary valuable traits (Perrett et al., 1998; Little et al., 2011). Overall, the available evidence points to a μ-opioid mechanism developed through evolution to mark the most valuable options and make them more desirable to us.

MOR activation is also involved in formation of partner preference, as indicated by recent evidence on social bonding in voles. Compared to the promiscuous meadow vole, the prairie vole has a significantly higher density of μ-opioid receptors in the nucleus accumbens shell, caudate and putamen. This is thought to contribute to species differences in social attachment behaviors (Inoue et al., 2013). A recent study showed that μ-opioid receptors were necessary for formation of lasting bonds between monogamous prairie vole mates (Burkett et al., 2011). Another study found that blocking μ-opioid receptors in the dorsomedial nucleus accumbens shell (dmNAcS) effectively impaired partner preference formation without affecting mating behavior. The authors proposed that MOR in the dmNAcS mediated pair bond formation by eliciting positive hedonic feelings associated with mating (Resendez et al., 2013). In other words, MOR activation may mediate a “hedonic marking” of available choices, making one partner stand out as preferable to the others.

The ability to experience MOR-mediated reward from social stimuli appears to be necessary for adequate social attachment. Genetic knock-out mouse pups lacking the μ-opioid receptor gene OPRM1 displayed diminished reward from mother-related cues as well as diminished distress in responses to separation from the mother, while distress responses to environmental threats remained normal (Moles et al., 2004). In other words, social reward is impaired without MOR, and both bond formation and the distress otherwise expressed when bonds are threatened by social separation are inhibited. The OPRM1 knockout model is now considered a comprehensive mouse model of autism spectrum disorder (Becker et al., 2014). The social reward deficiency in OPRM1 knockout mice mirrors the diminished reward responses to for instance cocaine and ethanol that have been observed in OPRM1 knock-out mice in other studies (Hall et al., 2001, 2004; Hall and Uhl, 2006), consistent with the notion of a shared MOR-driven mechanism underlying social motivation and addiction disorders (Panksepp et al., 1978; Machin and Dunbar, 2011). Research on natural variation of the μ-opioid receptor gene OPRM1 further supports a role for the MOR system both in modulating negative responses to social isolation, and in mediating the reward of social contact. Behaviorally, the OPRM1 118G-allele variant in humans and the OPRM1 77G in non-human primates are associated both with increased negative reactions to social separation *and* increased positive responses to social affiliation, i.e., enhanced social reward and rejection reactivity (Barr et al., 2008; Way et al., 2009; Higham et al., 2011; Troisi et al., 2011). For more details on OPRM1 and social behaviors, see Textbox 2.

Textbox 2 **The OPRM1 A118G/C77G genotype and affiliation.**

Single nucleotide polymorphisms (SNP) on the μ-opioid receptor gene OPRM1 occur naturally and result in functional variants of the receptor. In humans, the OPRM1 A118G SNP is one of the most studied SNPs on the OPRM1 gene, and an analog is found in rhesus macaque monkeys (OPRM1 C77G). While the exact molecular function of the OPRM 118/77 G-variant is still unclear (Zhang et al., 2005; Sia et al., 2008), the evidence for OPRM1 A118G/C77G modulation of social reward behaviors is building.

Compared to homozygous 77C-carriers, infant rhesus macaques carrying the 77G-allele exhibit stronger baseline attachment to their mothers, and after repeated mother-infant separation they display more intense distress responses to separation and greater contact time with mothers during reunion (Barr et al., 2008). Further, adult female rhesus macaques carrying the 77G-allele display increased maternal behaviors aimed at restraining their infant's movements to maintain physical proximity than C-allele homozygous females, suggesting that the G-allele is associated with greater maternal motivation to prevent separation (Higham et al., 2011).

In humans, the A118G polymorphism was associated with a greater dispositional sensitivity to social rejection, and with fMRI A118G carriers showed increased reactivity to social rejection in brain regions associated with processing of negative affect related to pain compared to A-homozygotes (Way et al., 2009). One study found that human adults carrying the OPRM1 G-allele self-reported higher tendencies to become engaged in affectionate relationships and experience more pleasure in social situations (Troisi et al., 2010). There is also some evidence that children with G-variant show stronger neural responses to faces with both angry and happy facial expressions compared to A-allele homozygotes (Bertoletti et al., 2012).

To sum up, the current evidence from human and rodent studies suggests that enhanced MOR system transmission increases appetitive reward responses for the preferred option available, both for non-social and social rewards. Disrupting the MOR system affects social reward processing and thereby the ability to form social bonds to a partner or parent. When the ability to experience the rewarding aspects of social interactions is impaired, being socially isolated or separated from a caregiver becomes less aversive. In contrast, high social reward responsiveness enhances separation distress.

### Separable μ-opioid modulation of positive and negative social affect

**Figure 3:**
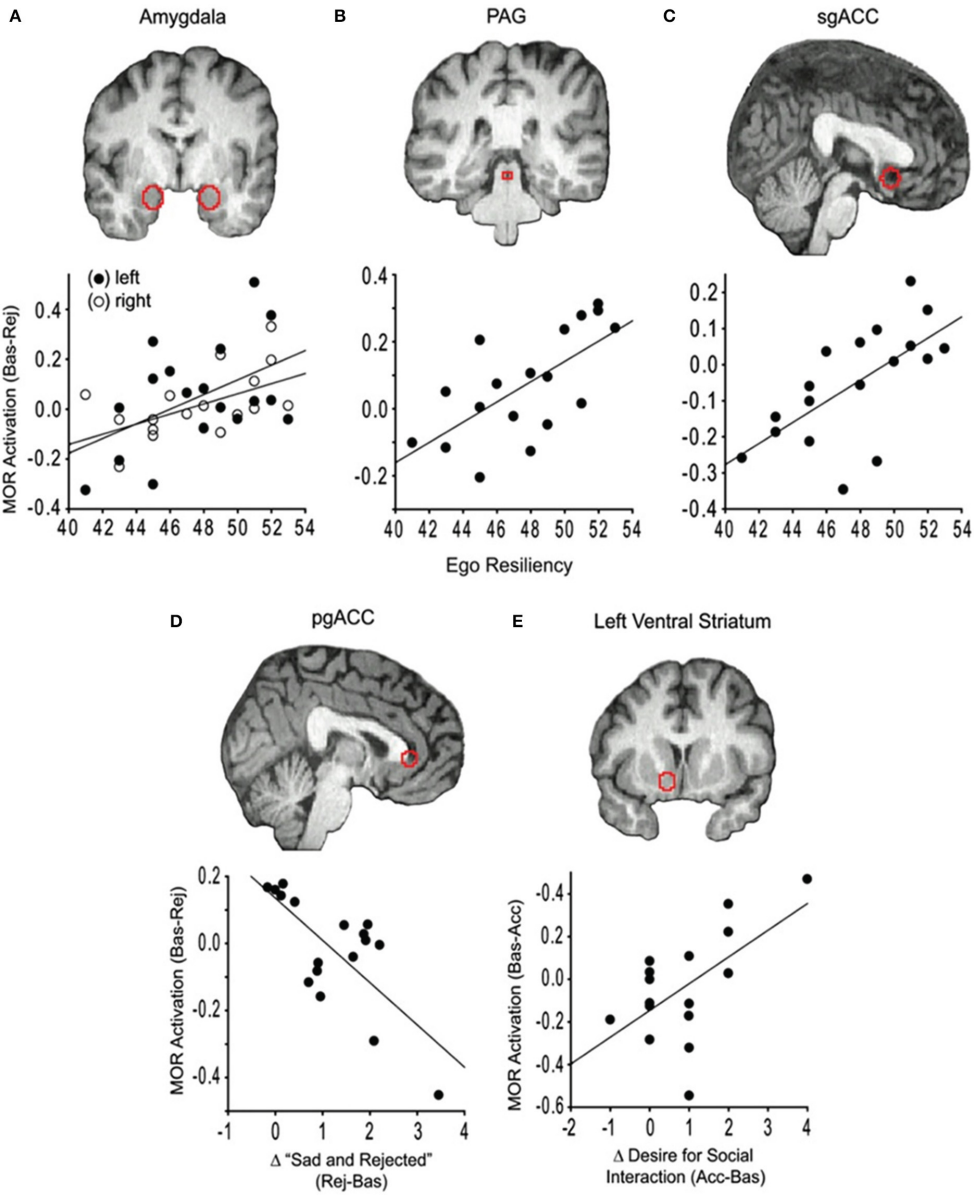
**MOR regulation of social affect in humans during social rejection and acceptance**. This figure, from Hsu et al. (2013), shows results from a PET study measuring endogenous MOR responses to conditions of social rejection or acceptance. During the Rejection state (receipt of social rejection signals from individuals in whom participants had indicated an interest), MOR activation in the amygdala **(A)**, periacqueductal gray (PAG) **(B)** and subgenual anterior cingulate cortex **(C)** correlated positively with measures of the trait ego resiliency, associated with feeling less sad and rejected during Rejection. This indicates that greater MOR activation in pain-modulating areas might be a neural substrate for resiliency, serving as a protective mechanism that makes the individual respond to social rejection with less distress. **(D)** Increased feelings of sadness and rejection were associated with lower activation of MOR in the pregenual anterior cingulate cortex during Rejection. **(E)** Increased social motivation correlated positively with increased MOR activation in the left ventral striatum during receipt of signals of social Acceptance. Reprinted from Hsu et al. (2013) by permission from Macmillan Publishers Ltd.

A recent molecular imaging study identified separable MOR mechanisms for modulation of negative and positive affective states caused by social interaction in humans. Hsu et al. (2013) let participants undergo a social feedback task in the scanner, where they were exposed to acceptance or rejection from persons they had rated as highly attractive and considered likely to reciprocate romantic interest (Figure 3). Compared to a neutral control condition, rejection led to increased MOR activation in the amygdala, periaqueductal gray (PAG), and right ventral striatum. High activity in these regions also predicted lower levels of sadness and feelings of rejection (Hsu et al., 2013), perhaps analogous to a coping mechanism for physical pain that is activated in stressful or threatening contexts (Fields, 2004).

Positive emotions of happiness and acceptance were increased during social acceptance, and this state change was accompanied by increases in MOR activation in the right anterior insula and left amygdala (Hsu et al., 2013). Similar MOR activation patterns were reported during positive mood induced by an amusing video clip (Koepp et al., 2009) and following amphetamine administration in humans (Colasanti et al., 2012). Interestingly, an increase in motivation to seek social contact was also observed in the acceptance condition, and this correlated positively with increased MOR activation in the ventral striatum (Hsu et al., 2013). In rats, stimulation of MOR in this region increase motivation for social interactions (Trezza et al., 2011b). Importantly, if motivational state (i.e., whether you are being socially accepted or rejected) alters the function of the MOR system in humans, could such state-dependence also explain the seemingly paradoxical, opposite effects of MOR manipulations in primates compared to juvenile and adult rodents?

### Does μ-opioid modulation of social motivation depend on state or species characteristics?

A large proportion of the studies investigating the role of the MOR system in affiliation have applied experimental paradigms where animals are subjected to a stressor such as separation from their mother, offspring, mate or social group in primates (Meller et al., 1980; Fabre-Nys et al., 1982; Keverne et al., 1989; Schino and Troisi, 1992; Martel et al., 1995; Graves et al., 2002) and rodents (Beatty and Costello, 1982; Panksepp et al., 1985; Siegel and Jensen, 1985, 1986; Vanderschuren et al., 1995c,d, 1996; Trezza and Vanderschuren, 2008a,b). Following the separation period, the MOR system is manipulated by central or peripheral administration of a MOR agonist or antagonist, and subsequent effects on distress-related behaviors, physiological stress responses, and social interactions are observed. Since social isolation is aversive to many social animals, experiments where the animal is taken away from its normal social setting before being reintroduced after a period of separation can be called *relief paradigms*.

Seeking social support is a central coping strategy for social animals facing some form of adversity (Cobb, 1976). It is useful here to distinguish distress from stress. The terms are often used interchangeably, yet distress implies another level of difficulty. When available coping mechanisms are insufficient to deal with stress, distress—which is characterized by more intense negative affect—ensues (NRC, 2008). Accordingly, animals that are more dependent on social comfort to handle stress should react with greater distress during isolation. This mechanism is illustrated by age-related differences in social distress. All mammalian infants rely on parental care to survive, and respond promptly to separation from the caregiver by emitting distress vocalizations and seeking physical proximity. The distress behaviors cease if comforting social contact is reinstated (Blumberg et al., 1992; Kalin et al., 1992; Hofer et al., 1993; Van Oers et al., 1998), and this comforting effect is thought to be mediated by MOR activation (Panksepp et al., 1980b; Kalin et al., 1988; Carden and Hofer, 1990b).

Later in the lifespan, species differences in social dependence become more apparent. Juvenile and adult rats respond to temporary social isolation with few signs of distress (Nelson and Panksepp, 1998) and no increase in stress hormone levels (Van Den Berg et al., 1999). Non-human primates on the other hand continue to display behavioral signs of distress upon social separation (Kalin, 1995; Rilling et al., 2001; Levine, 2005; Tardif et al., 2013). Depressive-like behavior similar to that observed in infant primates is displayed by adult primates separated from their family environment (Suomi et al., 1975), and physiological indications of stress such as increases in cortisol levels are observed in both juvenile and adult primates during social separation (Higley et al., 1992; Lyons et al., 1999; Ragen et al., 2013).

The discrepancy between MOR effects in rodents and primates could reflect a qualitative difference between social bonds in primates and rodents. Most rodents typically form transient bonds that serve to facilitate mating and nurturing of offspring, and that hinge on the hormonal context induced by copulation and parturition. They do not typically depend on enduring parental or social bonds for survival or successful offspring rearing (Broad et al., 2006). In contrast, primates are capable of forming long-lasting bonds even without such hormonal context (Broad et al., 2006). Primates live in highly complex social groups, on which they depend for protection from predators, as well as for cooperation on rearing of offspring and foraging for food (Dunbar, 2012). While social separation is a potential stressor for all social mammals, the occurrence and intensity of distress should reflect the potential harm from social isolation. Social bonds and interactions are valued higher in the more socially dependent primates, and it is possible that this is underpinned by an evolutionary shift in MOR system functioning—which in turn could explain why effects of MOR manipulation on social interactions following social separation are opposite for juveniles and adults of the two species.

However, considering the striking neurobiological correspondence between rodent and primate reward systems in general (Berridge and Kringelbach, 2008), and in μ-opioid effects on pain and social distress in particular (e.g., Eisenberger, 2012), we propose that the observed difference between these species rather reflects a difference in motivational state caused by affective responses to the experimental conditions.

## The SOMSOM: a model of state-dependent μ-opioid modulation of social motivation

**Figure 4:**
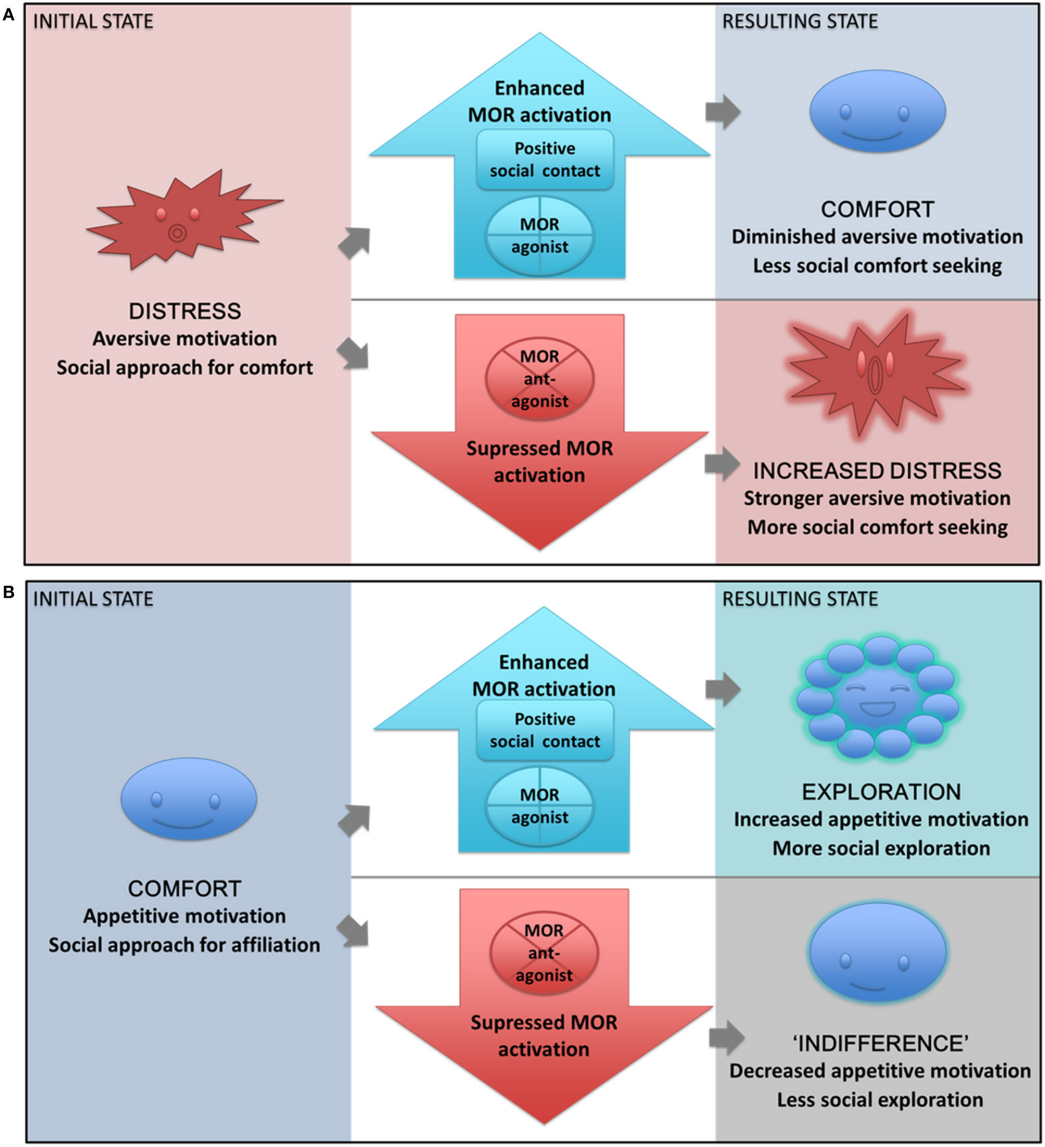
**The State-dependent μ-opioid Modulation of Social Motivation (SOMSOM) Model. (A)** During a separation distress state, motivation is predominately aversive, and social contact is sought out for comfort and relief. Positive social contact or MOR agonist treatment enhances MOR activation, thereby providing relief. The result is a comfort state where the need for social support is diminished, thus reducing social approach behaviors. When the MOR system instead is blocked with an antagonist, the resulting state is one of increased distress and enhanced aversive motivation. Social approach behaviors are thus increased, since the need for social support is greater. A similar reaction would be expected from further social isolation. **(B)** During a normal state of homeostatic balance, motivation is predominately appetitive, and social interaction is sought out for exploratory or affiliative purposes. Increased MOR transmission through MOR agonist treatment or positive social interaction leads to a state of exploration with increased appetitive motivation and increased social exploration and approach. If the MOR system instead is blocked by an antagonist, the resulting state is one of “indifference,” where appetitive social motivation is diminished and social approach is reduced.

Pain provides a powerful illustration of how the function of the MOR system can be modulated by motivational states. During high-pain states, large MOR agonist doses provide pain relief. Compared to non-pain states, acute pain is thought to reduce other common effects of opiate drugs, such as respiratory depression (Borgbjerg et al., 1996) and addiction potential (Ballantyne and Laforge, 2007), illustrating that the effect of exogenous μ-opioid manipulation is state dependent. According to the Motivation-Decision Model of Pain, the motivational context also determines the action of *endogenous* μ-opioids in the brainstem, such that pain signals can be either up- or down-regulated (Fields, 2006). If the context is dominated by a threat or pain signal, MOR deactivation can up-regulate that signal, amplify the experience of pain, and consequently facilitate rest and healing. In a context of potential high reward however, increased MOR activation may inhibit pain and thus enable reward-seeking in spite of risk. Conversely, pain inhibition can also facilitate escape from high threats. The purpose of this context-dependent mechanism is to enable decisions that maximize evolutionary benefits such as food or safety. We hypothesize that the MOR system affects social approach in a similarly context-dependent manner:

1. In a negative motivational state, social animals will seek out safe social contact as a mean of coping or comfort, i.e., to downregulate negative emotion through contact-mediated μ-opioid release. Stimulating MOR with opiates will then reduce social comfort seeking, because the need for comfort and endogenous MOR activation is diminished. Conversely, blocking MOR can enhance comfort seeking behaviors by blocking endogenous coping mechanisms.
2. In a positive motivational state on the other hand, social interaction can have functions beyond comfort, such as formation and maintenance of close social bonds, testing the boundaries of social hierarchies, or exploring possible new sex partners, and can thus be a source of fun, joy, pleasure—but also challenge and risk. As we have seen, the MOR system promotes many of these behaviors. Exogenous MOR stimulation should similarly increase the resources recruited for approach of social rewards, whereas an inhibition of social exploration is expected after MOR antagonism in this type of context (see Figure 4 for a schematic presentation of the model and its predictions).

When applying this model to the question of opioid modulation of affiliative behavior, we also draw on the ideas of Maslow, namely that motivation to meet higher needs is contingent on the prior fulfillment of more basic needs (Maslow, 1943). Specifically, if the μ-opioid system is needed to cope with pain or distress, this should be its primary function, and only after homeostatic equilibrium has been achieved would one expect MOR-mediated increases in social exploration. In both states, affiliative interaction is rewarding. During social distress, interaction is sought primarily for comfort, and achieving positive contact signals social support and aids down-regulation of negative affect. Comforting social contact is thus rewarding because it alleviates the unpleasant state, and the behaviors leading to social comfort are negatively reinforced. Otherwise, i.e., during homeostatic equilibrium, affiliative interaction is positively reinforced. When the interaction is sought primarily for fun or pleasure, affiliative behaviors indicate the establishment or maintenance of a social relationship, and are a source of joy. In the following section, we elaborate on our model by summarizing further evidence for state dependent μ-opioid modulation of social behaviors.

## μ-opioid modulation of social motivation for comfort or joy

The State-dependent μ-Opioid Modulation of SOcial Motivation (SOMSOM) model postulates that the diverging and paradoxical findings showing that MOR agonists in some cases *reduce* and in other cases *increase* social approach are due to very different motivational contexts. Although the intervention of social isolation or separation formed part of all the studies reviewed above, we argue that social separation leads to different motivational states depending on how much the animal relies on social contact for survival and emotion regulation. According to SOMSOM, the motivational state of the animal determines the effects of both agonism and antagonism of the MOR receptor, depending on whether the main motivation is to reduce distress or seek pleasure.

While studies of animals known to find social separation distressing, such as infant rodents; infant, juvenile and adult primates; and infants from other species, consistently show that the *comforting* effect social contact has on separation distress is blocked by MOR antagonism (see Section Introduction), blocking MOR with an antagonist is not found sufficient to induce separation distress in itself. Naltrexone failed to induce distress vocalizations in infant rats when they were allowed to remain together with their siblings (Carden et al., 1993). In primates, MOR antagonist treatment *reduced* maternal infant grooming in mothers who were never separated from their infant (Martel et al., 1993). These findings indicate that MOR blockade itself does not increase the need for social comfort or induce distress in a context where there is no prior stress. Instead, blockade of the MOR system in animals that remained unstressed attenuated social interaction.

Since social play generally occurs only when an animal is free from physiological and social stress, it provides a useful behavior for investigating MOR effects in the absence of distress (Loizos, 1967; Baldwin and Baldwin, 1976; Fagen, 1981; Siviy and Panksepp, 1985; Vanderschuren et al., 1995b; De Oliveira et al., 2003). By the time rats have become juveniles, social separation is no longer associated with distress (Nelson and Panksepp, 1998; Van Den Berg et al., 1999), and social separation leads to subsequent increases in social play interactions upon reunion (Panksepp, 1981). Based on the observation that play is suppressed by stress, we presume that the juvenile rats are in a state of appetitive motivation when they are released from brief isolation, and that they seek out social contact for joy (Panksepp and Burgdorf, 2003), and not primarily for consolation or to reduce distress (see also Textbox 1 for more details on social play). According to the SOMSOM model, MOR agonist treatment should lead to enhanced pleasure seeking when the animal is in a state of appetitive motivation. This is indeed what has been consistently found in pharmacological experiments of rodent social play (reviewed in Section Opposite μ-opioid System Modulation of Social Motivation in Primates and Rodents?). Further, social play was associated with strong increases in endogenous μ-opioid release in the rostral nucleus accumbens (Vanderschuren et al., 1995e), which is central to processing of other appetitive rewards (Berridge and Robinson, 1998; Trezza et al., 2011b).

Increased MOR transmission was hypothesized to enhance the reward value of social interactions (Vanderschuren et al., 1995a). This view is empirically supported by a study where a selective MOR agonist was injected directly into the nucleus accumbens of adolescent rats and observed to increase play behavior specifically, while injections of a specific MOR antagonist into this region prevented the development of social play-induced conditioned place preference (Trezza et al., 2011b). Exposing young rats to novel environments normally delays social play due to exploration of the surroundings (Vanderschuren et al., 1995b), but animals injected with low doses of morphine before being introduced to an unfamiliar cage immediately started social play interactions, which could indicate that MOR activation directs the attentional focus toward social rewards rather than exploring the environment (Trezza and Vanderschuren, 2008a). An alternative interpretation could be that the enhanced MOR transmission increases confidence (Panksepp et al., 1985), and thus reduces the need to check whether there are potential dangers present. Given its function in establishing social hierarchies and dominance relationships, engaging in social play involves a level of risk that could make it a more challenging rather than comforting activity (Poirier and Smith, 1974; Panksepp et al., 1985; Blumstein et al., 2013).

Few studies have investigated the effects of experimental MOR manipulation on play behaviors in primates, but the limited evidence suggests that the MOR system mediates social play in a similar manner to what is seen in rodents. In a study specifically designed to investigate MOR modulation of primate social play, marmoset juveniles living in a family group were injected with morphine, naloxone, or saline without prior social isolation-distress. Here, morphine specifically increased social play behaviors compared to both saline and naloxone, while naloxone led to slight, albeit not statistically significant, decreases in play behaviors compared to saline (Guard et al., 2002). Similarly, a study of long-tailed macaques that also were allowed to remain in their social context throughout the experiment, reported a decrease in social play after treatment with naloxone that approached statistical significance (Schino and Troisi, 1992). The weak effects of naloxone might indicate that other neurochemicals than μ-opioids are important for play behavior in primates. According to SOMSOM, MOR blockade during a state of distress should increase social motivation, specifically for comforting social interactions. As mentioned, social play is not a behavior usually sought out for comfort, so the increased social motivation during social distress would be expected to manifest in other behaviors. Indeed, a study where juvenile rhesus monkeys were socially isolated for 2 h found that the subsequent injection of naloxone, relative to saline, significantly decreased social play interactions when reunited with peers while contact with the mother increased (Martel et al., 1995). The authors hypothesized that the decrease in social play could reflect that MOR-blockade decreased social confidence and reduced the young monkeys' willingness to risk social play while increasing their need for social comfort (Martel et al., 1995).

In sum, and in line with SOMSOM, the evidence from rodent and primate studies shows that MOR activation in a context of positive motivation increased “appetitive” social play interactions, whilst MOR blockade reduced these behaviors, if to different degrees depending on species. The hypothesis that MOR activation enhances reward experienced during engagement in social play is in line with a large body of literature showing that MOR agonism enhances appetitive reward responses to food rewards (Berridge and Kringelbach, 2008; Difeliceantonio and Berridge, 2012; Mahler and Berridge, 2012).

## Conclusion

We started this review by presenting a paradox in the current literature on MOR modulation of social motivation, namely that MOR activation increases social motivation in rats, but reduces social motivation in primates. The experimental paradigms in most studies of rodents and primates include social separation followed by drug administration, and then social reunion. We reasoned that social separation would affect motivational state differently according to social dependency, such that for instance a socially dependent infant or an adult primate would seek comfort to regulate social distress. In contrast, an adult or juvenile rat would tolerate social separation and instead react to MOR activation with social exploratory behaviors. Importantly, the consistent correspondence between species in the reviewed literature on MOR modulation of reward processing, emotion regulation, and affiliation in humans, non-human primates, and rodents substantiates an interpretation of the initial paradox as a result of differences in motivational state caused by affective responses to the experimental conditions, rather than differences in neurobiology.

Building on the Motivation-Decision Model for opioid modulation of pain (Fields, 2006), and on the idea that motivation is dictated by a hierarchy of needs where more basic needs must be fulfilled before seeking to satisfy higher needs (Maslow, 1943), we have proposed a model for State-dependent μ-opioid Modulation of Social Motivation (SOMSOM). This model postulates that during a distress state, motivation is predominately aversive, and social contact is sought out for comfort and relief. Social comfort reduces distress at least in part via μ-opioid receptor (MOR) activation. Activation of MOR, elicited by either by social interaction or exogenous MOR agonist treatment, provides relief and diminishes the need for social support, thus reducing social approach behaviors. In contrast, during a normal state of homeostatic balance, motivation is predominately appetitive, and social interaction is sought out for explorative or affiliative purposes. Increased MOR transmission increases social exploration and approach of social rewards. In non-distressed states, decreased MOR transmission due to antagonist treatment reduces social approach.

The SOMSOM is supported by the reviewed evidence from a large literature of psychopharmacological studies in rats and a few key studies of non-human primates. When factors such as species and age differences in social dependency are taken into account, effects of pharmacological MOR manipulation on social approach behaviors are similar across species. We therefore hold that the SOMSOM predicts state-dependent MOR modulation of social motivation in humans. While the reviewed evidence from human studies show MOR system modulation of social motivation and separable MOR activation for positive and negative social affect, future studies should empirically test for state dependency in MOR modulation of human social motivation. Little is known about the role of the human MOR system in social bond formation and attachment. μ-opioid mechanisms form part of a complex concert of neurobiological processes modulating social behavior, and future studies should aim to disentangle interactions between the μ-opioid system and other neurochemical systems relevant for social reward such as κ-opioids and δ-opioids, oxytocin, dopamine and serotonin.

### Conflict of interest statement

The authors declare that the research was conducted in the absence of any commercial or financial relationships that could be construed as a potential conflict of interest.
